# A Genetic Association Study of Single Nucleotide Polymorphisms in FGFR1OP2/wit3.0 and Long-Term Atrophy of Edentulous Mandible

**DOI:** 10.1371/journal.pone.0016204

**Published:** 2011-01-19

**Authors:** Jaijam Suwanwela, Jaehoon Lee, Audrey Lin, T. Cemal Ucer, Hugh Devlin, Janet Sinsheimer, Neal R. Garrett, Ichiro Nishimura

**Affiliations:** 1 The Weintraub Center for Reconstructive Biotechnology, Division of Advanced Prosthodontics, Biomaterials and Hospital Dentistry, University of California Los Angeles (UCLA) School of Dentistry, Los Angeles, California, United States of America; 2 Division of Oral Biology and Medicine, University of California Los Angeles (UCLA) School of Dentistry, Los Angeles, California, United States of America; 3 Department of Prosthodontics, Faculty of Dentistry, Chulalongkorn University, Bangkok, Thailand; 4 Department of Prosthodontics, College of Dentistry, Yonsei University, Seoul, Korea; 5 School of Dentistry, University of Manchester, Manchester, United Kingdom; 6 Departments of Human Genetics and Biomathematics, David Geffen School of Medicine at the University of California Los Angeles (UCLA), Los Angeles, California, United States of America; University of Southern California, United States of America

## Abstract

**Background:**

After dental extraction, the external surface of alveolar bone undergoes resorption at various rates, and a group of patients develop excessive jawbone atrophy. Oral mucosa overlying the atrophied jawbone is unusually thin; therefore, we have hypothesized that excessive jawbone atrophy may be associated with abnormal oral mucosa contraction. FGFR1OP2/wit3.0, a cytoskeleton molecule initially identified in oral wound fibroblasts, has been shown to induce oral mucosa contraction after dental extraction. This study examined the genetic association between single nucleotide polymorphisms (SNPs) of FGFR1OP2/wit3.0 and excessive atrophy of edentulous mandible.

**Methods and Findings:**

First, the expression of FGFR1OP2/wit3.0 was determined in gingival tissues of 8 subjects before and after dental extraction. In situ hybridization revealed that all subject increased FGFR1OP2/wit3.0 expression in the post-operative oral mucosa tissues; however, significantly high levels of FGFR1OP2/wit3.0 were observed in 3 out of 8 subjects. In a separate study, 20 long-term edentulous subjects (66.4±9.4 years) were recruited. Tag-SNPs in the FGFR1OP2/wit3.0 allele were determined by Taqman-based polymerase chain reaction. The mandibular bone height was determined following the American College of Prosthodontists (ACP) protocol. Subjects with minor allele of rs840869 or rs859024 were found in the highly atrophied group by the ACP classification (Chi square test, p = 0.0384 and p = 0.0565, respectively; Fisher's Exact, p = 0.0515 and p = 0.2604, respectively). The linear regression analysis indicated a suggestive association between rs859024 and the decreased bone heights (Mann-Whitney, p = 0.06). The average bone height of the subjects with rs840869 or rs859024 minor alleles (10.6±3.2 mm and 9.6±3.2 mm, respectively) was significantly smaller than that of those subjects with the major alleles (14.2±4.5 mm, p<0.05).

**Conclusions:**

The patients with the minor allele of rs840869 or rs859024 were associated with excessive atrophy of edentulous mandible. This study may provide the basis for a genetic marker identifying susceptible individuals to develop jawbone atrophy after dental extraction.

## Introduction

Dental extraction is a common surgical treatment for dental caries and periodontal diseases. In a recent survey by the U.S. Center for Disease Control performed during 1999 to 2002, 8% of adults aged 20 years and older were edentulous, or have lost all of their natural teeth [1]. The prevalence of edentulism increases with age and reaches 25% of senior adults aged 60 years and older. The dental extraction wound normally heals uneventfully with bone formation in the tooth socket and bone resorption at the external surface of alveolar bone. As the result, the edentulous jawbone forms a saddle shape structure called the “residual ridge” [2], [3]. Since the 1950's, numerous clinical studies reported the loss of an unusually large volume and height of the residual ridge in some patients [2], [4], [5]. The reduction rate of residual ridge is most active during the first 6∼10 months after dental extraction. However, for some patients, active bone resorption persists even after the wound healing and results in the formation of excessive jawbone atrophy. In extreme cases, pathological bone fracture may occur in the atrophied mandible, which presents a significant challenge for patients and healthcare providers [6].

The underlying cause of residual ridge resorption has not been determined. Previous studies investigated the cause of edentulous jawbone atrophy in the context of bone physiology and pathology. For example, the systemic conditions associated with osteoporosis have been investigated as a causal factor of the residual ridge resorption and atrophied edentulous jawbones. It has been reported that ovariectomy in animal models negatively affects the healing of dental extraction wounds [7], [8], [9], [10], [11]. However, the correlation between osteoporosis and the development of edentulous jawbone atrophy has not been clearly demonstrated [12], [13], [14], [15]. Whereas trabecular bone in the edentulous jawbone appears to undergo remodeling processes at a comparative rate with other bones [16], [17], the separate osteoclastic activity is uniquely localized along the external surface of the residual ridge interfacing the oral mucosa [2], [17]. Therefore, bone resorption contributing to the residual ridge atrophy may be influenced by the edentulous oral mucosa. During the early healing period of dental extraction wounds, the gingival margins contract toward the center of the extraction socket and the epithelial integration is rapidly re-established [18], [19]. The newly regenerated epithelium forms a small central part of the edentulous oral mucosa [18], which is relatively thick with elongated rete pegs indicating acanthosis [20], [21]. On the contrary, the connective tissue thickness of the established residual ridge is found decreased in both denture wearers and non-denture wearers, whereas the collagen density is increased [22]. These findings collectively suggest that the oral mucosa undergoes continuous remodeling and the connective tissue contraction, which may result in a thin oral mucosa characteristically associated with the atrophic edentulous residual ridge.

After dental extraction, oral fibroblasts upregulate the expression of fibroblast growth factor receptor 1 oncogene partner 2/wound inducible transcript 3.0 (FGFR1OP2/wit3.0) [23], [24]. FGFR1OP2/wit3.0 is a cytoskeleton molecule and has been shown to polymerize and co-localize with stress fibers. Its over-expression accelerates the contraction of a fibroblast-populated floating collagen gel and full-thickness skin wounds in mice, while heterozygous null mutation of FGFR1OP2/wit3.0 decreased the migration rate of fibroblastic cells *in vitro* [25]. Thus, it has been postulated that FGFR1OP2/wit3.0 may, in part, regulate the accelerated oral wound contraction [26].

We have hypothesized that allelic variations of oral mucosa related molecules such, as FGFR1OP2/wit3.0, may be associated with excessive jawbone atrophy. In this study, the haplotype sensitive single nucleotide polymorphisms (SNPs) of FGFROP2/wit3.0 were evaluated in long-term edentulous subjects. We report here that the minor alleles of SNPs rs840869 and rs859024 of FGFR1OP2/wit3.0 may be associated with severely atrophied mandibles.

## Results

### Expression of FGFR1OP2/wit3.0 in human oral mucosa

**Figure 1:**
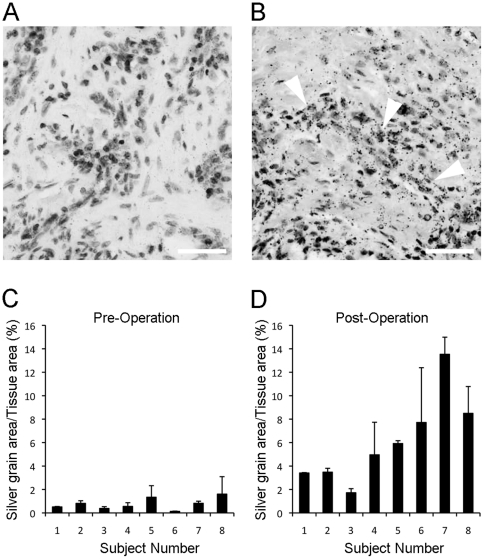
Expression of FGFR1OP2/wit3.0 in post-tooth extraction oral mucosa. **A**. In situ hybridization of FGFR1OP2/wit3.0 probe showed negative in human oral mucosa tissue prior to implant placement. Bar = 50 µm **B**. Oral mucosa tissue was harvested from the same subject when the implant was uncovered. The healed mucosa expressed positive hybridization of FGFR1OP2/wit3.0 probe in a cluster of fibroblasts (arrowheads). Bar = 50 µm **C**. In situ hybridization experiments in pre-operative oral mucosal tissue depicted constitutive FGFR1OP2/wit3.0 expression. **D**. The post-operative oral mucosal showed the increased expression of FGFR1OP2/wit3.0 in all subjects, albeit with significant variations.

Expression of FGFR1OP2/wit3.0 was evaluated in residual ridge oral mucosa harvested before and after dental extraction and immediate implant placement surgery by in situ hybridization. The FGFR1OP2/wit3.0 probe did not show strong hybridization in oral mucosa harvested before dental extraction (**Figure 1A**). Strong hybridization of FGFR1OP2/wit3.0 probe was, however, found in a cluster of fibroblastic cells in the equivalent oral mucosal tissues harvested after surgical wounding (**Figure 1B**). In some tissue samples, epithelial basal cells were also found to be positive for probe hybridization. The quantitative evaluation revealed that the pre-operative expression of FGFR1OP2/wit3.0 was a minimum and relatively uniform in most subjects (**Figure 1C**). All post-operative specimens showed an increased expression of FGFR1OP2/wit3.0 albeit with significant variations. It appears that 3 out of 8 subjects (37.5%) exhibited much higher expression levels of FGFR1OP2/wit3.0 (**Figure 1D**). The FGFR1OP2/wit3.0 expression data were further evaluated for age, sex and healing time; however, no significant trends associated with these factors were suggested.

### Mandibular residual ridge characterization

**Figure 2:**
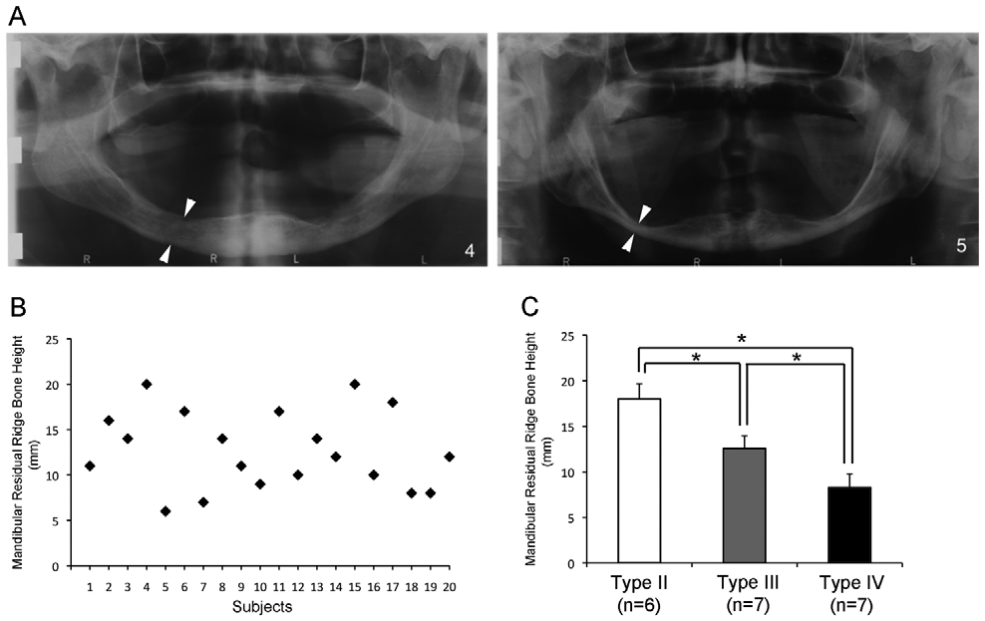
Characterization of mandibular edentulous ridge. **A**. Panoramic dental radiographs were used to determine the lowest residual ridge height (arrowheads) of edentulous mandible. Severe atrophy of edentulous mandible was observed in Subject #5 (right). **B**. Scatter plot of mandibular residual ridge heights indicated a uniform distribution between 6 to 20 mm. **C**. All subjects fell into one of the following ACP classifications: Type II (n = 6); Type III (n = 7); and Type IV (n = 7). Type II indicates mildly resorbed residual ridge, whereas Types III and IV are considered severely resorbed residual ridges. *P<0.05 by Student's t-test

In a separate study, a total of twenty long-term edentulous subjects (16 females and 4 males, average age 66.4±9.4 years old) were recruited for this study. All subjects were edentulous for more than 10 years and wore conventional complete dentures without dental implants. The ethnic composition of the edentulous subjects was 70% Whites (Western and Northern Europeans and Persians), 20% Asians (Continental Asians including Indians and Pacific Islanders), 5% Hispanic and 5% unreported. Panoramic dental radiographs were provided from all subjects, using which the bone height of the lowest vertical height of the edentulous mandible was measured following the protocol of the American College of Prosthodontists (ACP) [27] (**Figure 2A**). The mandibular bone height varied from 6 mm to 20 mm (**Figure 2B**). The ACP classification determines: Type I: residual ridge bone height of 21 mm or greater; Type II: residual ridge bone height of 16 to 20 mm; Type III: residual ridge bone height of 11 to 15 mm; and Type IV: residual ridge bone height of 10 mm or less. Of our edentulous subjects, 6 subjects were classified as Type II (18.0±1.7 mm); 7 subjects as Type III (12.6±1.4 mm); and 7 subjects as Type IV (8.3±1.5 mm). There was no subject in the Type I category.

### Non-synonymous SNPs of FGFR1OP2/wit3.0 in edentulous subjects

The coding sequence in exon 5 contains 2 non-synonymous SNPs. SNP rs1058701 substitutes A to C resulting in ^154^Glu to Asp; and SNP rs11613 substitute G to C resulting in ^155^Ala to Pro. It has been shown that the over-expression of FGFR1OP2/wit3.0 synthetic peptide carrying amino acid substitution by rs1058701 accelerates the rate of wound contraction in vitro and in vivo [25]. Thus, we first characterized the exon 5 in chromosomal DNA samples prepared from edentulous subjects by direct DNA sequencing. However, all subjects showed the homozygous wild type sequence and did not indicate the involvement of non-synonymous SNPs of exon 5 (**Table 1**).

**Table 1:** Frequencies of FGFR1OP2/wit3.0 SNPs in edentulous subjects (n = 20).

Rs #	Location in Chr12 ¶	SNP Sequence	Minor allele frequency	Hetero-zygosity	Methods	HWE**
rs2279351	27090550	A/C*	0.075	0.15	Taqman PCR	1.0000
rs840869	27093585	C/G*	0.200	0.30	Taqman PCR	1.0000
rs859024	27102857	A*/G	0.175	0.15	Taqman PCR	0.1372
rs2046937	27109655	C/G*	0.200	0.40	Taqman PCR	0.8387
rs1058701	27113513	A/C*	0.000	0.00	Direct Sequencing	1.0000
rs11613	27113514	C*/G	0.000	0.00	Direct Sequencing	1.0000
rs1051513	27115947	C*/T	0.225	0.25	Taqman PCR	0.4275
rs2129091	27119617	A/C*	0.600	0.30	Taqman PCR	0.1786

¶NCBI Reference Sequence: NC_000012.11;

*Minor allele listed in NCBI database;

**Hardy–Weinberg equilibrium of edentulous subjects.

### Taqman-based SNP genotyping of FGFR1OP2/wit3.0

Six SNPs rs2279351, rs840869, rs859024, rs2046937, rs1051513 and rs2129091 were further selected based on the assumptive tag-SNPs that were sensitive for haplotype constructs (NCBI Build 36). The genotype frequency of each of the SNPs was evaluated by Taqman-based PCR. These SNPs in the edentulous subjects were found within Hardy–Weinberg equilibrium and thus validated for further analyses (**Table 1**).

### SNP association with ACP Type II and Types III/IV

According to the ACP classification, Types I and II groups are considered as minimally or moderately resorbed residual ridges, whereas Types III and IV residual ridges are substantially or severely compromised due to jawbone atrophy [27]. Therefore, we evaluated the SNP association to Types III/IV as the compromised group and Type II as the “control” group. The minor allele frequency was relatively consistent in Type II and Types III/IV groups, except rs840869 and rs859024, whose minor alleles did not occur in Type II group (**Table 2** and **3**). The χ*^2^*test indicated that the presence of rs840869 minor allele in Types III/IV group was significant (p = 0.0384), whereas that of rs859024 was not significant but strongly suggestive (p = 0.0565) (**Table 2**).

**Table 2:** FGFR1OP2/wit3.0 SNP minor allele distribution and association with ACP Type II group and Types III/IV group. and UCLA archival DNA.

Rs #	Associated Allele	Type II, Types III/IV Ratio Counts	χ^2^	χ^2^ P value Type II v. Types III/IV
rs2279351	C	11∶1, 26∶2	0.017	0.8958
rs840869	G	0∶12, 8∶20	4.286	0.0384 **
rs859024	A	0∶12, 7∶21	3.636	0.0565 †
rs2046937	G	9∶3, 23∶5	0.268	0.6048
rs1051513	C	2∶10, 7∶21	0.335	0.5630
rs2129091	C	6∶6, 18∶10	0.714	0.3980

**p<0.05;

†p<0.1.

**Table 3:** FGFR1OP2/wit3.0 SNP minor allele distribution in edentulous subjects (n = 20) and UCLA archival DNA (n = 86) *.

Rs #	Associated Allele	Type II, Type III/IV Dominant minor allele frequency	Fisher's Exact P values Type II v. Types III/IV	UCLA Archive Dominant minor allele frequency
rs2279351	C	0.167, 0.143	1.000	0.244
rs840869	G	0.000, 0.500	0.0515 †	0.360
rs859024	A	0.000, 0.357	0.2604	0.279
rs2046937	G	0.500, 0.357	0.6424	0.279
rs1051513	C	0.333, 0.357	1.000	0.407
rs2129091	C	0.333, 0.214	0.6126	0.625

*Based on the presence of one or two copies of minor allele as the same effect (a dominant model);

†p<0.1.

Because of the small sample size, we next addressed non-parametric rather than parametric methods to assess significance. It has been reported that hetelogyzous null mutation of FGFR1OP2/wit3.0 was enough for mouse fibroblastic cells to significantly decrease the migration rate in vitro [25]. Therefore, we further evaluated the SNP data using the Fisher's Exact test by assuming that the presence of one or two copies of the minor allele should exhibit the same effect (a dominant model). The Fisher's Exact test indicated that the presence of rs840869 minor allele was significant in Types III/IV group at the 90% confidence level or p = 0.0515 (**Table 3**). The subjects with major alleles exhibited no specific distributions in ACP classifications.

### Linear regression analysis

The linear regression analysis and Mann-Whitney test were used to address if each SNP predicts the mandibular bone height. The dominant minor allele of SNP rs859024 showed a correlation to the edentulous bone height with the p value of 0.06 or at the 90% confidence level (**Table 4**). It was noted that the coefficient of rs859024 as well as rs840869 showed negative values, suggesting that having minor alleles in these SNP may contribute to the decrease in edentulous bone height (**Table 4**).

**Table 4:** Linear regression analysis and Mann-Whitney test for FGFR1OP2/wit3.0 SNP minor alleles and edentulous mandibular bone height *.

Rs #	F(1,18)	Prob>F	Adjusted R^2^	Root MSE	Coef. ± SE	Mann-Whitney: Prob>|z|
rs2279351	1.38	0.2558	0.0195	4.2146	3.10±2.64	0.1841
rs840869	2.97	0.1018	0.0941	4.0511	−3.27±1.90	0.1307
rs859024	4.12	0.0575 †	0.1409	3.945	−4.13±2.04	0.0596 †
rs2046937	1.02	1.02	0.0009	4.2544	1.96±1.94	0.3731
rs1051513	0.05	0.8242	−0.0526	4.3667	0.46±2.05	0.8736
rs2129091	1.20	0.2887	0.0102	4.2346	2.08±1.90	0.2527

*Based on the presence of one or two copies of minor allele as the same effect (a dominant model);

†p<0.1.

### SNPs rs840869 and rs859024 as predictors

**Figure 3:**
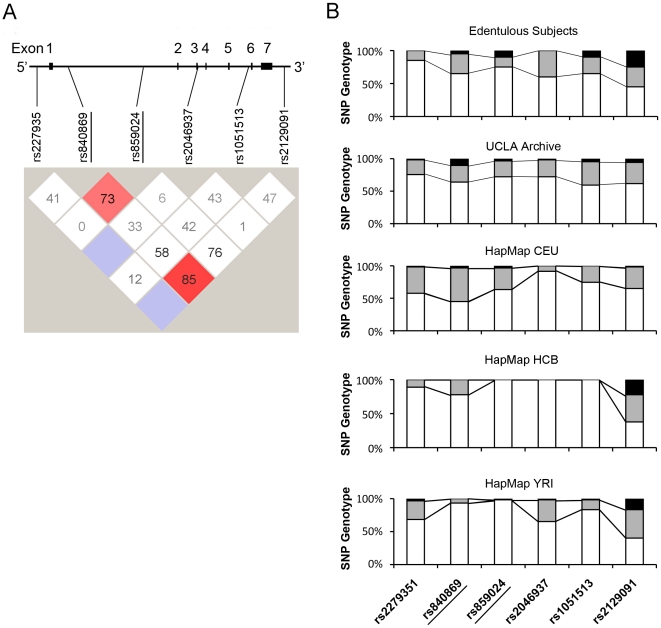
A. SNP-based haplotype analysis of FGFR1OP2/wit3.0 using the UCLA archival DNA samples. An association between rs840869 and rs859024 (underline) was indicated. **B**. Genotypes of FGFR1OP2/wit3.0 SNPs of edentulous subjects and UCLA archival DNA samples, as well as Western European descendents (CEU), Han Chinese in Beijing (HCB) and Sub-Saharan Africans (YRI) from the HapMap Phase III database. White bar: major allele homozygous; gray bar: heterozygous; and black bar: minor allele homozygous.

UCLA archival DNA samples were prepared from archived frozen pathological tissues with non-tumor diagnoses. Eighty-six DNA samples were used to evaluate the FGFR1OP2/wit3.0 SNPs. There was a suggestive linkage between rs840869 and rs859024 (**Figure 3A**). The genotype distributions of rs840869 and rs859024 of the edentulous subjects were similar to those of the UCLA archive DNA as well as to the haplotype database for Utah residents with Northern and Western European ancestry from the CEPH collection (HapMap CEU). It was noted that the genotype distributions of rs859024 were predominantly homozygous major allele in Han Chinese in Beijing (HapMap HCB); and furthermore, both rs840869 and rs859024 were indicated to be homozygous major alleles in Yoruba in Ibadan, Nigeria (HapMap YRI) (**Figure 3B**).

**Figure 4:**
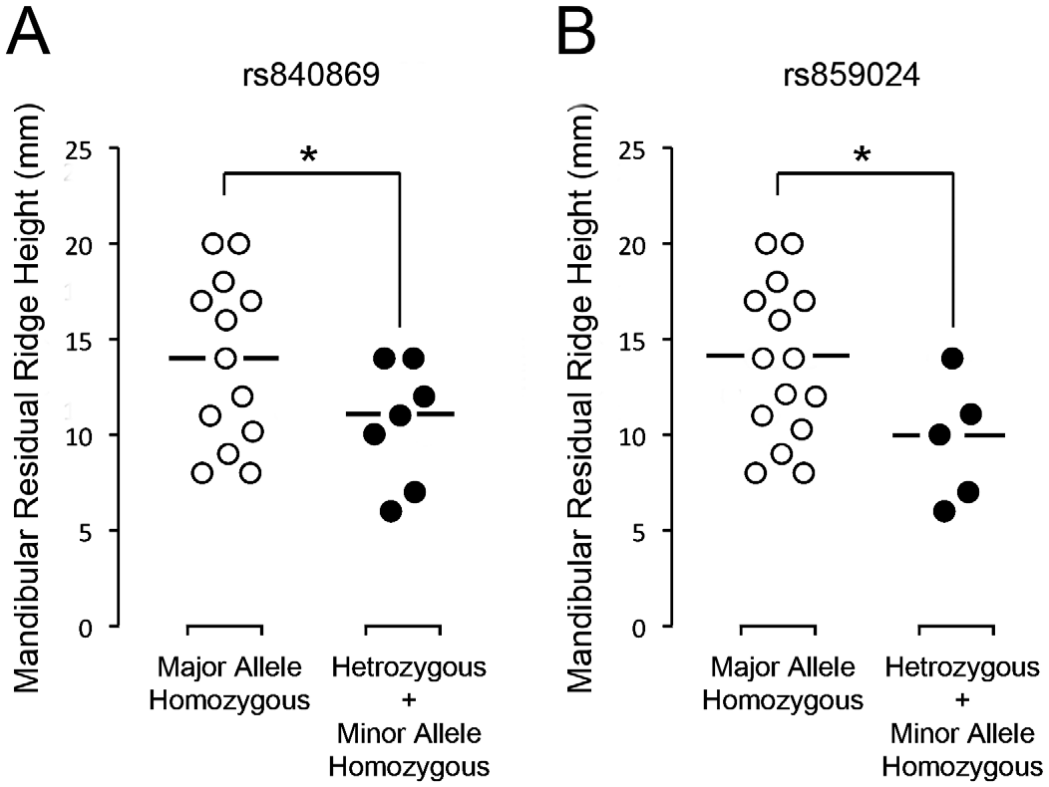
FGFR1OP2/wit3.0 SNPs rs840869 (A) and rs859024 (B) genotypes and mandibular edentulous ridge height. Subjects with the major allele homozygous of SNPs rs840869 and rs859024 (white circles) were found in Type II (n = 6) and Types III/IV (n = 6) groups with the median mandibular residual ridge height of 15.0 mm and the average height of 14.2±4.5 mm. Subjects with heterozygous or minor allele homozygous of rs840869 and rs859024 (black circles) were found only in Types III/IV group with median mandibular edentulous ridge height of 11 mm and 10 mm, respectively, and the average height of 10.6±3.2 mm and 9.6±3.2 mm, respectively. * P<0.05.

The average mandibular bone height of subjects containing at least 1 minor allele of rs840869 or rs859024 was 10.6±3.2 mm and 9.6±3.2 mm, respectively, which was significantly shorter than those with homozygous major SNP alleles with the average height of 14.2±4.5 mm (p<0.05) (**Figure 4A and 4B**). The dominant minor allele of rs840869 was found in 36.0% of UCLA archival DNA, which was increased to 50.0% in the Types III/IV edentulous group, whereas the Type II edentulous group did not carry this minor allele (**Table 2** and **3**). The s859024 dominant minor allele was also not found in the Type II group, but was found in 27.9% and 35.7% of UCLA archival DNA and the Types III/IV group, respectively (**Table 3**).

## Discussion

This study demonstrated that the SNP genotype of the FGFR1OP2/wit3.0 allele may predict the severity of post-dental extraction jawbone atrophy. In particular, SNPs rs840869 and rs859024 showed a possible association with the severely resorbed edentulous mandible (**Figure 4A and 4B**). Edentulous subjects with dominant minor allele of rs840869 and/or rs859024 fell into the ACP Type III/IV group and none of these subjects were found in Type II group (**Table 2** and **3**). The ACP classification Types I and II refer to minimally to moderately compromised edentulous ridges, whereas Types III and IV refer to substantially or severely compromised edentulous ridges [27]. Therefore, individuals carrying dominant minor allele of rs840869 and/or rs859024 may have an increased risk of developing atrophied edentulous mandible.

Surgical removal of tooth creates a large void in alveolar bone or the socket, which undergoes a series of wound healing events. The bone socket initiates new bone apposition, while the external surface of alveolar bone is subjected to localized bone resorption [28], resulting in the generation of a saddle-shaped residual ridge [29]. Previous studies of atrophic edentulous jaw primarily focused on bone-related factors such as osteoporosis with unclear associations [13], [15], [30]. The candidate gene selected in the present study is not involved in bone but rather in wound healing of oral mucosa. Whereas adult skin wounds heal with significant scar formation, wounds in oral mucosa heal with less scar formation. FGFR1OP2/wit3.0 was isolated as a differentially expressed transcript in oral fibroblasts after dental extraction wounding [23]. FGFR1OP2/wit3.0 peptide possesses the characteristics of a cytoskeleton molecule, and the increased expression of this molecule has been shown to significantly enhance cell migration and wound contraction [24], [25]. The Online Mendelian Inheritance in Man (OMIM; http://www.ncbi.nlm.nih.gov/omim/608858, last accessed on 12/4/2010) identified a (12;8)(p11;p11p22) insertion in a 75-year-old male who presented with a T-cell lymphoblastic lymphoma that progressed rapidly to acute myelogenous leukemia. The gene fragment of 12p11 encoding exon 1 to 4 of FGFR1OP2/wit3.0 was inserted in chromosome 8p22, resulting in the ligand-independent fusion peptide of FGFR1OP2-Fibroblast Growth Factor Receptor 1 (FGFR1) [31]. Because the reciprocal FGFR1-FGFR1OP2 transcripts were not detected, it is likely that this insertion mutation should result in the null mutation of FGFR1OP2/wit3.0. To date, dental and oral information of this patient is not available.

SNPs rs840869 and rs859024 are “tag-SNPs” representing a region of chromosomal DNA with high linkage disequilibrium. We have selected tag-SNPs for this study because they are useful in identifying the potential genetic association with wider spectra. It must be noted, however, that tag-SNPs themselves may not necessarily contribute to the functional variations. Our haplotype analysis indicated an association between rs840869 and rs859024 albeit at a marginal significance (**Figure 3A**). Therefore, it may be suggested that any SNPs located in the haploblock(s) tagged by rs840869 and/or rs859024 may possess the functional relevancy. To date, 272 SNPs have been identified in the FGFR1OP2/wit3.0 allele, of which 78 SNPs are located between rs840869 and rs859024 (**Table S1**). These “candidate SNPs” are found in the first intron, which often carries cis-acting elements regulating gene transcription. Although the mechanism of FGFR1OP2/wit3.0 transcription has not been well understood, the regulatory cis-acting elements with different efficiencies might be a possible functional variation affecting the level of FGFR1OP2/wit3.0 in the residual ridge soft tissue.

The sustained expression of FGFR1OP2/wit3.0 has been observed in rat oral soft tissue even 60 days after dental extraction (unpublished data). However, the long-term expression in the human residual ridge has not been established. The major reduction of the height and width of jawbone occurs during this initial dental extraction wound healing of 6–10 months, followed by the slow but continuous bone resorption. The present study demonstrated that dental extraction increased the expression of FGFR1OP2/wit3.0 in human oral fibroblasts during 3 to 8 weeks of wound healing following surgery (**Figure 1B**); however, the rate of FGFR1OP2/wit3.0 expression significantly varied among subjects (**Figure 1D**). It is tempting to speculate that the first intron genotype variation might contribute, in part, to different FGFR1OP2/wit3.0 gene transcription activities at least during the initial wound healing stage.

The homozygous major allele genotype of rs840869 and rs859024 was found in the majority of UCLA archival DNA samples (64.0% and 72.1%, respectively). The edentulous subjects with the major allele genotypes showed a wide range of edentulous residual ridge heights (**Figure 4A and 4B**). It has been postulated that a number of environmental factors may contribute to the individual variations such as time post-extraction and duration of denture wearing [2], [5]. All subjects were edentulous for over 10 years; however, it is conceivable that the environmental confounding factors may have influenced the variation in their bone heights. On the contrary, the subjects with a dominant minor allele showed highly resorbed jawbones. Because similar environmental factors are likely to have affected all subjects, the compromised residual ridge in this group may, in part, be contributed by a mechanism related to the rs840869 and rs859024 genotypes.

The haplotype database indicated that SNP genotypes of rs840869 and rs859024 were diverse among Western European descendents, Han Chinese and Sub-Saharan Africans (**Figure 3B**). From the available ethnicity data, the UCLA archival DNA samples were composed of 89.4% Whites (Western, Eastern and Northern Europeans and Persians), 6.4% Asians (Continental Asians including Indians and Pacific Islanders) and 4.3% Blacks. SNP genotypes of edentulous subjects were generally in agreement with those of UCLA archival DNA samples. The possible involvement of ethnic diversity in the SNP genotypes in FGFR1OP2/wit3.0 was not evaluated in this study. It was further noted that the SNP rs2129091 genotype of the edentulous subjects was significantly different from that of UCLA archival DNA samples (Fisher's Exact test, p = 0.021) (**Table 3**; **Figure 3B**). The minor rs2129091 allele may suggest a susceptibility marker for edentulism; however further investigations are needed.

This study has demonstrated the first indication of a genetic basis to atrophic jawbone resorption. The outcome of this study immediately suggests a novel genetic diagnostic method to identify patients predisposed to severe atrophy of jawbone structure. Such a genetic diagnosis may provide a patient's healthcare team with an objective basis to different treatment recommendations. One of the limitations of the study is the small numbers of subjects that were available to us. Therefore, these results must be interpreted as preliminary and require replication. However, FGFR1OP2/wit3.0 may provide a novel clue to understanding the pathological mechanism of post-dental extraction atrophy of jawbone leading to the targeted preventive and therapeutic modalities.

## Materials and Methods

### Ethics Statement

All research involving human participants were performed in accordance with ethical standards of the responsible committees on human experimentation and with the Helsinki Declaration of 1975, as revised in 2008. HD was the principal investigator of the Manchester study entitled “Expression of Unique Proteins in Wounded Oral Mucosa (LREC study number 02/BR/255),” which was approved by the Bury and Rochdale Local Ethics Committee, Manchester, UK. IN was the principal investigator of the University of California at Los Angeles (UCLA) study entitled “Genetic Markers for Accelerated Bone Loss in the Lower Jaw (UCLA IRB #05-11-101)”, which was approved by the UCLA Institutional Review Board, Los Angeles, CA, USA. The written informed consent was obtained from all subjects prior to participating in these studies.

### Expression of FGFR1OP2/wit3.0 in human oral tissues

In the Manchester study, 8 volunteer subjects were recruited from those scheduled for dental implant treatment. After written consent was obtained, biopsy specimen was obtained from the healthy mucosal tissues just prior to tooth extraction for subjects scheduled for delayed immediate implant placement surgery (pre-operation). Three to 8 weeks after dental extraction and at the time of implant uncovering, the second oral mucosal biopsy specimen was obtained from the extraction site (post-operation). The oral mucosal specimens were kept in biopsy specimen containers (10% buffered formalin) and processed for conventional paraffin wax histology. All tissue samples were labeled with anonymously assigned identification numbers.

In-situ hybridization was carried out using cDNA probes encoding Exon 5 of FGFR1OP2/wit3.0 following the established method [32]. The silver grains were counted under a light microscope, and custom software was used to determine an average area of silver grains in a given “area of interest”. For each specimen, this was repeated in order to determine an average number of silver grains per histological section. The number of silver grains outside of the tissue were counted in each specimen as background hybridization and subtracted from the tissue data to give an “adjusted” silver grain count. The slide was viewed under ×20 magnification. On average 0.15 mm^2^ of tissue was examined for each subject (range 0.08 to 0.31 mm^2^). Each image underwent processing to increase the contrast response (black top hat) and thresholding by selecting pixels with a similar intensity. The observer was blinded during the assessment as to the identity of the slide, and the code was broken only after completion of the experiment. The mean silver grain density was recorded (percentage of the area covered by silver grains) using a Quantimet Q600 image analyzer linked to a Leika DMRB microscope (Leica UK, Milton Keynes, UK).

### Edentulous subjects

In the UCLA study, unrelated healthy adult volunteer subjects with edentulous maxilla and mandible and exhibited difficulty in denture treatment were recruited from UCLA Dental Clinics. Those subjects the met the following criteria were included in the study: 1) complete edentulism for 10 years or longer; 2) no treatment for alveolar bone augmentation; 3) no known systemic conditions that could affect bone conditions such as osteoporosis, hyperparathyroidism, and others; 4) no history of neoplastic lesions in the oral cavity; and 5) presenting a panoramic dental radiograph of less than 1 year old. After informed consent was obtained, oral examination was performed to confirm their edentulous condition. Subjects with significant midfacial damage, leading to severe distortion or absence of one or more jaws were excluded. A total of 20 volunteer subjects were enrolled in this study.

### Measurement of mandibular residual ridge height

The panoramic dental radiograph of each subject was digitized with the anonymously assigned identification number, and stored in a secured computer with password protection. Following the American College of Prosthodontists (ACP) classification, the lowest vertical height of the edentulous mandible was measured. The ACP classification was determined as: Type I: residual ridge bone height of 21 mm or greater; Type II: residual ridge bone height of 16 to 20 mm; Type III: residual ridge bone height of 11 to 15 mm; and Type IV: residual ridge bone height of 10 mm or less [27].

### FGFR1OP2/wit3.0 SNPs

Each edentulous subject was asked to rinse his or her mouth with 20 ml of alcohol-free mouthwash solution for 30 seconds and then expectorate the solution into a 50 ml sample tube [33]. The mouthwash solution was stored in 4°C for no longer than 12 hours. DNA samples were prepared from the exfoliated oral tissues in the mouthwash solution using a conventional method (QIAamp DNA Blood Mini Kit, Qiagen, Valencia, CA).

Separately, 86 DNA samples were prepared from archived frozen pathological tissues without neoplasmic diagnoses provided by the Department of Pathology & Laboratory Medicine, the David Geffen School of Medicine at UCLA. All DNA samples with anonymously assigned identification number were stored in a locked deep freezer (−80°C).

To identify the presence of the Exon 5 non-synonymous SNPs, aliquots of each DNA sample was subjected to direct sequencing with oligonucleotide primers flanking Exon 5 of FGFR1OP2/wit3.0. Separately, 6 SNPs were selected from the assumptive tag SNPs (SNPbrowser Software, Applied Biosystems, Carlsbad, CA). DNA samples were subjected to Taqman-based SNP evaluation using commercially available primer/probe cocktails (Applied Biosystems).

### Data analyses

First, we determined whether individual variants were in equilibrium at each locus in the population (Hardy–Weinberg equilibrium). The association of SNPs with the mandibular jawbone atrophy was further analyzed by assuming that the presence of one or two copies of the minor allele should exhibit the same effect (a dominant model).

The subjects were separated in 2 groups: the less atrophied group (ACP Type II; n = 6) and the significantly atrophied group (ACP Types III/IV; n = 14). The comparison of SNPs between Type II and Types III/IV groups was evaluated based on the dominant model using the Fisher's Exact test. The comparison of SNPs of UCLA archival DNA to Type II and to Types III/IV groups was also evaluated by the χ*^2^*test and Fisher's Exact tests.

The χ*^2^*test and Mann-Whitney test were used for the quantitative trait analysis of each SNP with the dominant model. Bone height was used as the outcome and each SNP as a potential predictor of bone height. The coefficient and its standard error were calculated by linear regression analysis.

We further examined Lewontin's D’ (|D’|) and the linkage disequilibrium (LD) coefficient r^2^ for all pairs of biallelic loci. Haploview version 3.2 (Whitehead Institute for Biomedical Research, Cambridge, MA, USA) was used for the structure of the LD block and association analysis [34].

## Supporting Information

Table S1 The complete list of SNPs in the FGFR1OP2/wit3.0 allele
(DOCX)

## References

[1] Beltrán-Aguilar ED, Barker LK, Canto MT, Dye BA, Gooch BF (2005). Surveillance for dental caries, dental sealants, tooth retention, edentulism, and enamel fluorosis—United States, 1988–1994 and 1999–2002.. MMWR Surveill Summ.

[2] Jahangiri L, Devlin H, Ting K, Nishimura I (1998). Current perspectives in residual ridge remodeling and its clinical implications: a review.. J Prosthet Dent.

[3] Van der Weijden F, Dell'Acqua F, Slot DE (2009). Alveolar bone dimensional changes of post-extraction sockets in humans: a systematic review.. J Clin Periodontol.

[4] Carlsson GE (1998). Clinical morbidity and sequelae of treatment with complete dentures.. J Prosthet Dent.

[5] Kingsmill VJ (1999). Post-extraction remodeling of the adult mandible.. Crit Rev Oral Biol Med.

[6] Nasser M, Fedorowicz Z, Ebadifar A (2007). Management of the fractured edentulous atrophic mandible.. Cochrane Database Syst Rev.

[7] Li X, Nishimura I (1994). Altered bone remodeling pattern of the residual ridge in ovariectomized rats.. J Prosthet Dent.

[8] Hsieh YD, Devlin H, McCord F (1995). The effect of ovariectomy on the healing tooth socket of the rat.. Arch Oral Biol.

[9] Pereira MC, Zecchin KG, Campagnoli EB, Jorge J (2007). Ovariectomy delays alveolar wound healing after molar extractions in rats.. J Oral Maxillofac Surg.

[10] Shoji K, Elsubeihi ES, Heersche JN (2010). Effects of ovariectomy on turnover of alveolar bone in the healed extraction socket in rat edentulous mandible.. Arch Oral Biol.

[11] Luvizuto ER, Dias SM, Queiroz TP, Okamoto T, Garcia IR (2010). Osteocalcin immunolabeling during the alveolar healing process in ovariectomized rats treated with estrogen or raloxifene.. Bone.

[12] Ward VJ, Stephens AP, Harrison A, Lurie D (1977). The relationship between the metacarpal index and the rate of mandibular ridge resorption.. J Oral Rehabil.

[13] Klemetti E, Vainio P, Lassila V, Alhava E (1993). Trabecular bone mineral density of mandible and alveolar height in postmenopausal women.. Scand J Dent Res.

[14] Bollen AM, Taguchi A, Hujoel PP, Hollender LG (2004). Number of teeth and residual alveolar ridge height in subjects with a history of self-reported osteoporotic fractures.. Osteoporos Int.

[15] Kribbs PJ, Smith DE, Chesnut CH (1983). Oral findings in osteoporosis. Part II: Relationship between residual ridge and alveolar bone resorption and generalized skeletal osteopenia.. J Prosthet Dent.

[16] Kribbs PJ, Smith DE, Chesnut CH (1983). Oral findings in osteoporosis. Part I: Measurement of mandibular bone density.. J Prosthet Dent.

[17] Devlin H, Sloan P, Luther F (1994). Alveolar bone resorption: a histologic study comparing bone turnover in the edentulous mandible and iliac crest.. J Prosthet Dent.

[18] Simpson HE (1969). The healing of extraction wounds.. Br Dent J.

[19] Turck D (1965). A Histologic Comparison of the Edentulous Denture and Non-Denture Bearing Tissues.. J Prosthet Dent.

[20] Watson IB, MacDonald DG (1982). Oral mucosa and complete dentures.. J Prosthet Dent.

[21] Schroeder HE, Amstad-Jossi M (1986). Epithelial differentiation at the edentulous alveolar ridge in man. A stereological study.. Cell Tissue Res.

[22] Krajicek DD, Dooner J, Porter K (1984). Observations on the histologic features of the human edentulous ridge. Part II: Connective tissue.. J Prosthet Dent.

[23] Sukotjo C, Abanmy AA, Ogawa T, Nishimura I (2002). Molecular cloning of wound inducible transcript (wit 3.0) differentially expressed in edentulous oral mucosa undergoing tooth extraction wound-healing.. J Dent Res.

[24] Sukotjo C, Lin A, Song K, Ogawa T, Wu B (2003). Oral fibroblast expression of wound-inducible transcript 3.0 (wit3.0) accelerates the collagen gel contraction in vitro.. J Biol Chem.

[25] Lin A, Hokugo A, Choi J, Nishimura I (2010). Small cytoskeleton-associated molecule, fibroblast growth factor receptor 1 oncogene partner 2/wound inducible transcript-3.0 (FGFR1OP2/wit3.0), facilitates fibroblast-driven wound closure.. Am J Pathol.

[26] Lin A, Hokugo A, Nishimura I (2010). Wound closure and wound management: A new therapeutic molecular target.. Cell Adh Migr.

[27] McGarry TJ, Nimmo A, Skiba JF, Ahlstrom RH, Smith CR (1999). Classification system for complete edentulism. The American College of Prosthodontics.. J Prosthodont.

[28] Araujo MG, Lindhe J (2005). Dimensional ridge alterations following tooth extraction. An experimental study in the dog.. J Clin Periodontol.

[29] Fickl S, Zuhr O, Wachtel H, Bolz W, Huerzeler M (2008). Tissue alterations after tooth extraction with and without surgical trauma: a volumetric study in the beagle dog.. J Clin Periodontol.

[30] Horner K, Devlin H (1998). The relationships between two indices of mandibular bone quality and bone mineral density measured by dual energy X-ray absorptiometry.. Dentomaxillofac Radiol.

[31] Grand EK, Grand FH, Chase AJ, Ross FM, Corcoran MM (2004). Identification of a novel gene, FGFR1OP2, fused to FGFR1 in 8p11 myeloproliferative syndrome.. Genes Chromosomes Cancer.

[32] Devlin H, Hoyland J, Newall JF, Ayad S (1997). Trabecular bone formation in the healing of the rodent molar tooth extraction socket.. J Bone Miner Res.

[33] Mulot C, Stucker I, Clavel J, Beaune P, Loriot MA (2005). Collection of human genomic DNA from buccal cells for genetics studies: comparison between cytobrush, mouthwash, and treated card.. J Biomed Biotechnol.

[34] Barrett JC, Fry B, Maller J, Daly MJ (2005). Haploview: analysis and visualization of LD and haplotype maps.. Bioinformatics.

