# Supplementary material for: A Genetic Association Study of Single Nucleotide Polymorphisms in FGFR1OP2/wit3.0 and Long-Term Atrophy of Edentulous Mandible
Source: PLoS One. 2011 Jan 19;6(1):e16204. doi: 10.1371/journal.pone.0016204 (PMC3023796; doi:10.1371/journal.pone.0016204)
Supplement: Table S1 — The complete list of SNPs in the FGFR1OP2/wit3.0 allele (DOCX) [file pone.0016204.s001.docx]

| Chr. Position | dbSNP rs# | dsSNP allele | Note |
| --- | --- | --- | --- |
| 27089484 | rs114765785 | A/C |  |
| 27089540 | rs2306852 | A/G |  |
| 27089806 | rs11611563 | A/C |  |
| 27090308 | rs75924113 | G/T |  |
| 27090550 | rs2279351 | A/C | Tag-SNP used in this study |
| 27090587 | rs75780371 | C/T |  |
| 27090653 | rs61923397 | C/T |  |
| 27090774 | rs117637388 | A/C |  |
| 27090871 | rs4397951 | C/T |  |
| 27090930 | rs116387372 | A/G |  |
| 27090942 | rs61923398 | A/G |  |
| 27090944 | rs2345261 | A/G |  |
| 27091011 | rs113645979 | A/G |  |
| 27091104 | rs78054962 | C/T |  |
| 27091242 | rs12314170 | C/T |  |
| 27091275 | rs61923399 | C/G |  |
| 27091455 | rs115146680 | A/G |  |
| 27091457 | rs115084512 | G/T |  |
| 27091483 | rs11542284 | C/G |  |
| 27091544 | rs7979780 | A/G |  |
| 27091830 | rs35463167 | -/A |  |
| 27092032 | rs116905813 | A/T |  |
| 27092072 | rs113949938 | A/G |  |
| 27092124 | rs12316065 | A/G |  |
| 27092126 | rs11048778 | A/G |  |
| 27092181 | rs78516792 | G/T |  |
| 27092249 | rs117733441 | A/T |  |
| 27092417 | rs77879463 | A/G |  |
| 27092663 | rs116181994 | A/G |  |
| 27092871 | rs57151752 | A/G |  |
| 27092893 | rs111798266 | C/T |  |
| 27092971 | rs708161 | A/G |  |
| 27093110 | rs78102086 | A/G |  |
| 27093216 | rs16931498 | C/G |  |
| 27093347 | rs112549054 | C/G |  |
| 27093421 | rs73084621 | A/T |  |
| 27093478 | rs117915524 | G/T |  |
| 27093569 | rs76788395 | C/T |  |
| 27093585 | rs840869 | C/G | Tag-SNP used in this study |
| 27093625 | rs74978372 | A/T |  |
| 27094033 | rs115895303 | A/T |  |
| 27094390 | rs11830459 | C/T |  |
| 27094666 | rs78354188 | G/T |  |
| 27094731 | rs74672503 | G/T |  |
| 27094736 | rs78142848 | C/T |  |
| 27094879 | rs11048779 | C/T |  |
| 27094927 | rs114363686 | G/T |  |
| 27094969 | rs117800624 | C/T |  |
| 27095034 | rs34450455 | -/G |  |
| 27095376 | rs34373854 | -/A |  |
| 27095618 | rs112408900 | G/T |  |
| 27095711 | rs7954107 | G/T |  |
| 27095996 | rs117008822 | C/G |  |
| 27096098 | rs116097678 | A/G |  |
| 27096112 | rs77295009 | C/T |  |
| 27096208 | s112183278 | -/AA |  |
| 27096230 | rs57552082 | C/G |  |
| 27096233 | rs10842816 | C/G |  |
| 27096317 | rs74729366 | A/G |  |
| 27096336 | rs112354263 | C/T |  |
| 27096404 | rs80277854 | -/A |  |
| 27096409 | rs76907714 | -/A |  |
| 27096410 | rs58116369 | -/A |  |
| 27096507 | rs708162 | A/C |  |
| 27096587 | rs111394361 | A/G |  |
| 27096770 | rs77079154 | A/G |  |
| 27097020 | rs34689200 | -/G |  |
| 27097077 | rs3912842 | A/C |  |
| 27097512 | rs1070769 | A/G |  |
| 27097698 | rs75713800 | C/G |  |
| 27097727 | rs75144190 | C/T |  |
| 27097927 | rs7296867 | A/T |  |
| 27098034 | rs75394455 | G/T |  |
| 27098202 | rs1098528 | C/T |  |
| 27098319 | rs12300607 | A/C |  |
| 27098322 | rs112965570 | A/G |  |
| 27098427 | rs117803427 | A/C |  |
| 27098531 | rs12300776 | C/T |  |
| 27098809 | rs74728377 | -/AAAA |  |
| 27098817 | rs116795676 | A/G |  |
| 27098822 | rs67784352 | A/G |  |
| 27098900 | rs7979884 | G/T |  |
| 27098935 | rs57444779 | -/TT |  |
| 27099150 | rs11610153 | A/G |  |
| 27099283 | rs73084627 | C/G |  |
| 27099482 | rs115508781 | A/T |  |
| 27099488 | rs112633966 | C/G |  |
| 27099559 | rs79305696 | A/T |  |
| 27099677 | rs7955335 | A/G |  |
| 27099715 | rs11048780 | A/C |  |
| 27099719 | rs77071133 | A/G |  |
| 27099783 | rs11048781 | C/G |  |
| 27100001 | rs12302815 | C/G |  |
| 27100024 | rs73084630 | C/T |  |
| 27100282 | rs79195111 | A/G |  |
| 27100350 | rs115730874 | C/T |  |
| 27100426 | rs113118136 | A/G |  |
| 27100492 | rs864552 | A/T |  |
| 27100864 | rs12582211 | A/G |  |
| 27100892 | rs7959342 | A/G |  |
| 27101044 | rs73084631 | C/G |  |
| 27101183 | rs73088763 | A/G |  |
| 27101391 | rs114164527 | A/T |  |
| 27101530 | rs115135172 | A/G |  |
| 27101631 | rs116698293 | C/T |  |
| 27101642 | rs10842817 | C/T |  |
| 27101797 | rs75595121 | C/T |  |
| 27101806 | rs79701518 | A/G |  |
| 27102051 | rs112565245 | A/G |  |
| 27102124 | rs7973402 | A/C |  |
| 27102154 | rs7973588 | C/T |  |
| 27102172 | s12422617 | A/G |  |
| 27102586 | rs840868 | A/G |  |
| 27102605 | rs111408624 | G/T |  |
| 27102797 | rs77761025 | A/G |  |
| 27102857 | rs859024 | A/G | Tag-SNP used in this study |
| 27102950 | rs61923400 | A/G |  |
| 27103058 | rs113283051 | A/G |  |
| 27103136 | rs12228996 | A/T |  |
| 27103144 | rs2450850 | G/T |  |
| 27103146 | rs2450851 | -/G/GTT/T |  |
| 27103147 | rs34480417 | -/GTT |  |
| 27103162 | rs5797205 | -/TTTTT |  |
| 27103163 | rs71784330 | -/TTTTT |  |
| 27103282 | rs1185210 | C/G |  |
| 27103291 | rs1183779 | A/G |  |
| 27103313 | rs1184839 | C/G |  |
| 27103314 | rs113752672 | C/G |  |
| 27103405 | rs61923401 | C/T |  |
| 27103462 | rs1186001 | A/T |  |
| 27103489 | rs1186002 | A/G |  |
| 27103545 | rs117182108 | C/G |  |
| 27103551 | rs77918688 | C/T |  |
| 27103588 | rs79716120 | A/G |  |
| 27103736 | rs59272806 | G/T |  |
| 27103747 | rs10842818 | C/T |  |
| 27103898 | rs112027037 | A/G |  |
| 27103913 | rs10842819 | A/G |  |
| 27103945 | rs11048782 | A/G |  |
| 27103979 | rs73290202 | G/T |  |
| 27104093 | rs12229415 | C/T |  |
| 27104094 | rs11048783 | C/T |  |
| 27104409 | rs7132565 | C/T |  |
| 27104937 | rs117471424 | C/T |  |
| 27105019 | rs76048423 | C/T |  |
| 27105021 | rs34270981 | -/T |  |
| 27105037 | rs76684479 | -/TTT |  |
| 27105040 | rs57050256 | -/T |  |
| 27105040 | rs11048784 | A/T |  |
| 27105041 | rs115567583 | A/T |  |
| 27105042 | rs76905688 | A/T |  |
| 27105187 | rs117438079 | C/T |  |
| 27105221 | rs79303714 | C/G |  |
| 27105253 | rs12303825 | A/G |  |
| 27105311 | rs113256283 | A/G |  |
| 27105410 | rs16931510 | C/T |  |
| 27105411 | rs77810249 | C/T |  |
| 27105624 | rs77876591 | G/T |  |
| 27105667 | rs113583622 | C/G |  |
| 27105710 | rs1872192 | G/T |  |
| 27105748 | rs58830215 | C/T |  |
| 27105924 | rs61618590 | A/G |  |
| 27106113 | rs7295544 | C/T |  |
| 27106157 | rs708163 | A/G |  |
| 27106201 | rs34664034 | -/A |  |
| 27106202 | rs10714749 | -/A |  |
| 27106202 | rs36095097 | -/A |  |
| 27106211 | rs11287790 | -/A |  |
| 27106222 | rs58462576 | -/A |  |
| 27106222 | rs71069286 | -/A |  |
| 27106242 | rs117173100 | C/G |  |
| 27106251 | rs58329571 | -/GGTAAG |  |
| 27106252 | rs75003999 | G/T |  |
| 27106540 | rs7296043 | A/G |  |
| 27106808 | rs75688976 | A/G |  |
| 27107013 | rs73292204 | C/T |  |
| 27107148 | rs116940418 | A/G | synonymous: 19[L][L] |
| 27107268 | rs2129092 | A/G |  |
| 27107564 | rs11611754 | G/T |  |
| 27107716 | rs117398317 | A/G |  |
| 27108261 | rs12581929 | C/T |  |
| 27108496 | rs117884518 | A/G |  |
| 27108674 | rs114709723 | C/T |  |
| 27108797 | rs75364406 | A/T |  |
| 27108875 | rs58705648 | C/G |  |
| 27108963 | rs57254536 | -/T |  |
| 27108964 | rs111752257 | A/T |  |
| 27109039 | rs117147567 | C/T |  |
| 27109216 | rs11048785 | A/T |  |
| 27109251 | rs11048786 | A/G |  |
| 27109404 | rs4036421 | A/T |  |
| 27109406 | rs4036422 | A/C |  |
| 27109413 | rs71437311 | -/ATAT |  |
| 27109424 | rs71919040 | -/TA |  |
| 27109433 | rs67397655 | -/AT |  |
| 27109434 | rs5797206 | -/AT |  |
| 27109435 | rs36098229 | -/AT |  |
| 27109603 | rs114028106 | A/G |  |
| 27109655 | rs2046937 | A/G | Tag-SNP used in this study |
| 27109693 | rs115900629 | A/G |  |
| 27109776 | rs112845177 | -/T |  |
| 27109874 | rs77320563 | A/G |  |
| 27109878 | rs116155061 | A/G |  |
| 27109888 | rs117576062 | C/T |  |
| 27109904 | rs60312461 | A/T |  |
| 27109936 | rs78319398 | G/T |  |
| 27110001 | rs73292207 | C/T |  |
| 27110023 | rs840895 | C/T |  |
| 27110027 | rs58111600 | C/G |  |
| 27110135 | rs16931514 | C/T |  |
| 27110357 | rs112325643 | A/G |  |
| 27110558 | rs78308909 | A/G | non-synonymous: 93[R][H] |
| 27110915 | rs76716352 | A/G |  |
| 27111064 | rs11048787 | C/T |  |
| 27111192 | rs113560306 | G/T |  |
| 27111321 | rs840896 | A/G |  |
| 27111542 | rs74368859 | A/G |  |
| 27111840 | rs73088769 | A/G |  |
| 27111890 | rs71437312 | -/A |  |
| 27111966 | rs1166773 | C/G |  |
| 27111983 | rs1166772 | C/G |  |
| 27112640 | rs115658591 | C/T |  |
| 27112757 | rs114883831 | C/T |  |
| 27112859 | rs115723127 | A/G |  |
| 27113513 | rs1058701 | A/C | non-synonymous: 154[E][D] |
| 27113514 | rs11613 | G/C | non-synonymous: 155[A][P] |
| 27113658 | rs117491617 | A/G |  |
| 27113682 | rs76601721 | G/T |  |
| 27113952 | rs118178465 | C/T |  |
| 27113953 | rs78418583 | G/T |  |
| 27113995 | rs12581901 | C/T |  |
| 27114015 | rs17407618 | A/G |  |
| 27114073 | rs76840860 | A/T |  |
| 27114076 | rs114612886 | G/T |  |
| 27114078 | rs75758513 | A/C |  |
| 27114179 | rs12580822 | A/G |  |
| 27114324 | rs12580815 | C/G |  |
| 27114821 | rs80343260 | A/G |  |
| 27114990 | rs116425301 | A/G |  |
| 27115133 | rs16931524 | A/G |  |
| 27115203 | rs111552868 | G/T |  |
| 27115395 | rs6487582 | C/T |  |
| 27115455 | rs75856909 | C/G |  |
| 27115704 | rs17486118 | C/T |  |
| 27115947 | rs1051513 | C/T | Tag-SNP used in this study |
| 27116238 | rs76561184 | A/G |  |
| 27116440 | rs80250225 | A/G |  |
| 27116745 | rs12314911 | C/G |  |
| 27117299 | rs114243261 | A/G |  |
| 27117382 | rs11048788 | C/G |  |
| 27117426 | rs80189711 | A/G |  |
| 27117589 | s78951705 | A/C | synonymous: 191[R][R] |
| 27117662 | rs118150543 | A/G | non-synonymous: 215[N][S] |
| 27117690 | rs114547360 | C/T |  |
| 27118497 | rs111796365 | A/T |  |
| 27118613 | rs900743 | G/T |  |
| 27118615 | rs112380753 | A/C |  |
| 27118844 | rs116041150 | A/G |  |
| 27118938 | rs12818194 | A/T |  |
| 27118972 | rs12819730 | A/T |  |
| 27119066 | rs12824645 | A/T |  |
| 27119067 | rs12823559 | G/T |  |
| 27119384 | rs114344031 | C/G |  |
| 27119474 | rs118070818 | A/T |  |
| 27119475 | rs79079534 | A/T |  |
| 27119476 | rs74835021 | G/T |  |
| 27119490 | rs80002985 | G/T |  |
| 27119491 | rs118053480 | G/T |  |
| 27119492 | rs61923403 | G/T |  |
| 27119495 | rs117108258 | G/T |  |
| 27119502 | rs116043123 | A/C |  |
| 27119617 | rs2129091 | A/C | Tag-SNP used in this study |
